# Phenotype and Molecular Characterizations of 30 Children From China With *NR5A1* Mutations

**DOI:** 10.3389/fphar.2018.01224

**Published:** 2018-10-30

**Authors:** Yanning Song, Lijun Fan, Chunxiu Gong

**Affiliations:** ^1^Beijing Children’s Hospital, Capital Medical University, National Center for Children’s Health, Beijing, China; ^2^Beijing Key Laboratory for Genetics of Birth Defects, Beijing Children’s Hospital, Capital Medical University, Beijing, China

**Keywords:** *NR5A1* mutation, novel mutation, phenotype and genotype, prader grade, steroli cell

## Abstract

**Background:** Patients harboring *NR5A1* mutations have a wide spectrum of phenotypes.

**Objective:** To investigate the phenotype of patients with *NR5A1* gene mutations from a 30 Chinese patient cohort.

**Methods:** We reported the clinical features of children with *NR5A1* gene mutations and compared them between two groups of patients with social genders of male (boys group) and female (girls group).

**Results:** Thirty patients with *NR5A1* mutations ranging from 2 months to 17 years of age were studied. There were 11 boys and 19 girls who were identified when they visited the hospital. The patients were verified as having testes without a uterus and ovaries by B-mode ultrasound. There was no difference between boys and girls in terms of the Prader stage (*p* = 0.086), but the position of the testes was higher in girls than in boys (*p* = 0.013). The patients’ average height is −0.43 SDS according to the normal boys’ height with SDS (while their average target height was 0.07 SDS). However, there was no such difference between boys and girls (*p* > 0.05). Although the basal LH and post-hCG testosterone (T) levels were not different (*p* > 0.05), but the basal FSH level, LH/FSH ratio, and INHB level were decreased in girls (*p* = 0.002; *p* = 0.001; *p* = 0.006). All of the mothers of the patients reported to have normal pregnancies. We found 24 patients (80%) with *de novo* mutations in the *NR5A1* gene; 5 patients had inherited mutations from their mothers, and one inherited from the father. Only the mothers of patients 16 and 18 showed premature ovarian failure at the time of reporting. Among 26 disease associated mutations, 14 novel mutations that have been reported the first time and p.R87C is the most common Among the other 12 had had been reported,the p.R313C is the most common.

**Conclusion:** Patients with 46, XY *NR5A1* mutations presented a wide spectrum of external genitalia characteristics and severe Sertoli cell impairment. The p.R87C and p.R313C mutations appeared to be common (10%) in this group, and 14 new mutations were identified, improving our understanding the genotype phenotype correlations.

## Introduction

Disorders of sex development (DSDs) are defined as congenital conditions in which the development of chromosomal, gonadal, or phenotypic sex is abnormal (Ono and Harley, 2013). DSDs have been divided into three groups: Sex chromosome DSDs, 46, XX DSDs and 46, XY DSDs. The incidence of complete gonadal dysgenesis is estimated to be 1:80000 in newborns (Michala et al., 2008). One gene, *NR5A1* gene located on chr 9q33.3, has emerged play a major role as a common genetic cause in 10–20% of 46, XY DSD cases in the last few years (Suntharalingham et al., 2015; Takasawa et al., 2017), it encodes steroidogenic factor-1 (SF-1). SF-1 is a key regulator of steroidogenesis and reproductive development that controls several steps of adrenal and gonadal development. It stimulates the expression of several genes required for the development and maintenance of the male differentiation cascade. This gene also regulates the expression of *LHCGR* and the steroidogenic enzymes *STAR, CYP11A1*, and *CYP17A1* in Leydig cells, which are required for testosterone biosynthesis. *NR5A1* also increases the expression of insulin-like polypeptide 3 (INSL3) (Schimmer and White, 2010), which regulates testicular descent and is a survival factor for male germ cells in adults. The phenotypical spectrum encompasses from hypospadias (Tantawy et al., 2014), ambiguous genitalia, such as a hypoplastic phallus (Werner et al., 2015), to a complete external female appearance (Allali et al., 2011). The *NR5A1* gene has one nontranslated exon (exon 1) and six other coding exons (exon 2–7). SF1 has two zinc finger DNA-binding domains (DBDs), a ligand-binding domain (LBD), two functional activation domains (AF-1 and AF-2), an accessory region and a hinge region. The DBD contains a core with two Cys4 zinc finger motifs and a highly conserved Ftz-F1 box motif that is potentially involved in interactions with DNA (Parker and Schimmer, 1997).

Achermann et al. (1999, 2002) initially reported in 1999 and again in 2002 that heterozygous mutations in the *NR5A1* gene could cause adrenal insufficiency for patient with 46, XY severe testicular dysplasia. However, subsequent reports found only gonad dysfunction without adrenal insufficiency (Mallet et al., 2004; Philibert et al., 2007; Reuter et al., 2007; Kohler et al., 2008, 2009; Tajima et al., 2009). To date, only 4 given heterozygous mutations (p.G35D p.G35E, p.R92Q, and p.R255L) can cause adrenal insufficiency combined with gonadal dysfunction have been reported (Achermann et al., 1999, 2002; Orekhova et al., 2017).

Patients with 46, XY DSD present with normal AMH levels without a uterus or fallopian tubes (Coutant et al., 2007; Brandt et al., 2013), or have low concentrations of AMH and detectable Müllerian structures on B-mode ultrasound (Brandt et al., 2013). Some patients with low levels of AMH at birth but without apparent Müllerian structures have also been reported (Coutant et al., 2007; Kohler et al., 2009). In most cases, testosterone level is low during the neonatal period. Therefore, the phenotype has been known ranging from ambiguous genitalia to female external genitalia at birth (Tantawy et al., 2014; Yagi et al., 2015). However, there are also reports stating that patients with normal testosterone concentrations at birth or even at puberty may show spontaneous pubertal progression or obvious virilization. This phenomenon suggests that the function of Leydig cells is sufficient for some patients. Patients showing persistently elevated FSH concentrations and low INHB and AMH concentrations have progressive gonadal failure that appears to occur with age, especially affecting the Sertoli cells; this finding is in accordance with the results of another study in which they followed up several patients younger than 30 years of age who presented with progressive gonadal dysgenesis after adolescence (Fabbri et al., 2014; Yagi et al., 2015; Werner et al., 2017).

Recently, in addition to causing 46, XY DSDs and adrenal dysfunction, missense mutations in *NR5A1* were identified as a cause of 46, XX testicular/ovotesticular disorders of sexual development (Baetens et al., 2017; Igarashi et al., 2017; Takasawa et al., 2017). While a genotype–phenotype relationship has not been established to date, approximately 120 *NR5A1* mutations have been documented in the Human Gene Mutation Database^1^. Nonsense mutations are the most common. In this study, we evaluated the clinical features and genotypes of 30 Chinese Han patients with 46, XY DSDs.

## Materials and Methods

### Patients

Patients ranging in age from 2 months to 17 years with various degrees of ambiguous external genitalia and *NR5A1* gene mutations were recruited from 30 unrelated families. All of the patients had been confirmed to have 46, XY karyotype. The clinical diagnoses in the patients with *NR5A1* mutations were based on incomplete virilization features, such as hypospadias, microphallus, cryptorchidism, clitoromegaly and complete female external genitalia. All of the patients also underwent *AR* and *SRD5A2* genetics analysis to exclude androgen insensitivity syndrome and 5α-reductase type 2 deficiency, respectively. The informed consent for participation in the study were documented. The research protocol was approved by the Ethics Committee of Beijing Children’s Hospital, Capital Medical University.

### Clinical Information

The same pediatric endocrinologist performed these physical examinations and assessments. These information included age, social gender, chief complaint, family history, height, weight, facial features, clitoris/penis length, testicular position, urethral and vaginal meatus, electrolyte levels, and liver and kidney function, etc. Pituitary hormones and T concentrations were measured, and B-mode ultrasound or MRI was used to examine the patients’ kidneys, adrenal glands, pelvic gonads and ducts. **T**hese patients were grouped based on their social gender of either male or female. The phenotype, hormones and gene mutations were compared between the two groups. The outcomes of the results led the individuals to undergo selective surgery or medical treatment for boys or girls for adaption to the reared gender.

### Clinical Features: Meatus and Testes Position and Classification

(i) Prader classification included the following values (Moshiri et al., 2012): Prader stage 0–1 = 1, Prader stage 2 = 2, Prader stage 3 = 3, Prader stage 4 = 4, and Prader stage 5 = 5. A Prader stage ≤ 3 was considered a severe phenotype. (ii) The testis position was classified with the following scores: abdominal = 1, inguinal = 2, labia = 3, and scrotum = 4. The lower the score was, the more serious the position was considered to be.

Hormone measurement was determined as follows. First, we examined the basal T concentrations. If T was at a prepubertal concentration, an hCG stimulation test was performed; namely, an injection of 15.00 IU of hCG per day was administered for 4 consecutive days. Peripheral blood samples were obtained after 12 h. T, FSH, LH, ACTH, and Cor were evaluated by radioimmunoassay techniques. MAGLUMI^®^2000. AMH was used for electrochemiluminescence, and INHB was evaluated by an ELISA assay.

### Molecular Analysis

The *NR5A1*, *AR*, and *SRD5A2* genes were detected by the Beijing Key Laboratory for Genetics of Birth Defects and then confirmed by Sanger sequencing from January 2010 to July 2017. The sequencing data were analyzed by the authors.

### Evaluation of Variant Pathogenicity

The genomics data are based on comparison with the NCBI reference sequence NM_004959. PolyPhen-2^2^, SIFT^3^ and the ACMG guidelines were used to predict the impact of the identified mutations on protein function.

### Statistical Analysis

We compared the clinical Prader stages, testes position values, basal T levels, T levels after hCG stimulation and LH/FSH ratios. In this study, the normally distributed values are described as the mean ± standard deviation and were compared by *t*-tests. The nonnormally distributed data are described by the median and were compared with the Mann-Whitney U test. A 2-tailed *p-*value < 0.05 was considered statistically significant for all analyses. All statistical analyses were conducted using SPSS Statistics version 17.0.

## Results

### Clinical Features

There were 30 patients with 46, XY DSDs who had *NR5A1* mutations. The patients’ gonadal tissues were all testes without residual Müllerian or ovotesticular structures. Considering gender identity at birth, there were 19 girls and 11 boys. Thirteen of the girls presented with an inguinal mass, and the remaining 6 had obvious virilization at the first visit (older than 10 years with an obvious “Adam’s apple,” hoarse voice, and clitoris virilization). The Prader stage of the external genitalia ranged from 0 to 4. One of the 11 boys had a micropenis, and the others had hypospadias (Prader stage of 2–5). There was no difference between genders when comparing Prader stages (*p* = 0.086), but the position of the testes was higher in girls than in boys (*p* = 0.013). The average height of all subjects was −0.43 SDS according to the normal boys’ height standard (their average target height was 0.07 SDS), but there was no difference between boys and girls (*p* > 0.05). The bone ages were not clearly different. All mothers of the patients reported a normal pregnancy. The mothers of patients 16 and 18 carried the same mutations as the probands and showed delayed puberty and POI (menarche at 14 and 18 years and menopause at 40 and 36 years). Two sisters of patient 18 carried the same mutation and had delayed puberty, and two nephews (sons of the elder sister’s) showed 46, XY hypospadias. The other patients’ family histories were negative. The clinical characteristics of the patients are shown in Tables 1, 2.

**Table 1:** Clinical data of 30 children with *NR5A1* mutations ID.

	Height (SDS)	PG	TP	Basal						P-hCG	AMH	INHB	Family History
							
				LH (mIU/ml)	FSH (mIU/ml)	T (ng/dl)	LH/FSH	ACTH (pg/ml)	Cor (ug/dl)	T (ng/dl)			
1	−0.33	3	2	0.1	2.62	<20	0.04	27	9.1	183.7	23	83	N
2	0.19	2	2	0.07	1.94	10.08	0.04	12	7.2	300			N
3	−1.12	2	2	6.51	31.9	99.1	0.2	14	8.5	526			N
4	0.67	3	3	2.33	9.88	<20	0.24	15	7	120			N
5	−1.43	3	2	0.42	1.04	<20	0.4	24	9.8	231	1.43	32.5	N
6	−2.48	2	2	0.21	3.17	<20	0.07	42	11.8	168	23	99	N
7	−1.9	4	2	19.2	67	289	0.29	27	8.5	N/A			N
8	−0.22	3	2	0.22	4	<20	0.06	23	10.2	101			N
9	1.24	2	1	14.2	66	130	0.22	22	7.3	147			N
10	0.37	0	2	1.43	9.08	<20.0	0.16	24	11	219			N
11	0.31	4	2	4.25	22.2	121	0.19	23	9.6	632			N
12	−1.49	1	2	2.76	36.1	71.8	0.08	15	14.2	469	2.19	20.18	N
13	−0.33	2	2	0.1	2.13	<20	0.05	25	8.3	<20	7.89	33.6	N
14	0.11	0	3	3.67	30.1	86	0.12	18	8	579	23	59	N
15	−0.33	2	2	0.07	1.94	10.08	0.04	20	10.1	197	23	57.2	N
16	0.73	3	1	14.5	49.2	171	0.29	21	12.7	N/A	1.73	2.46	46, XX POI
17	0.31	2	2	3.95	28.4	209	0.14	18	16.5	N/A			N
18	−0.6	4	4	22.24	46.43	123.5	0.48	29	9.1	N/A	0.19	7.56	46, XX POI + 46, XX DSD
19	−0.93	2	2	4	23.4	100	0.17	34	6.9	N/A			N
20	0.75	3	4	0.46	2.83	61	0.16	11	5	595	23	88	N
21	−1.5	5	2	0.1	1.3	20	0.08	18	13	164	10.96	89.2	N
22	−1.21	4	2	3.54	17.2	81.5	0.21	19	8	N/A			N
23	−2.27	3	4	N/A	N/A	N/A	–	13	11.2	N/A			N
24	0.37	3	4	2.87	17.6	141	0.16	20	5.2	312			N
25	−0.68	3	3	0.1	3.2	<20	0.31	14	6.5	<20	23	39.3	N
26	−0.87	3	2	1.23	2.38	116	0.52	33	15.8	274	23	21.5	N
27	0.95	3	4	0.83	1.7	20	0.49	19	14.8	157	6.09	52.65	N
28	0.03	4	4	1.24	1.46	20	0.85	17	6.9	321	23	188.5	N
29	−0.1	3	4	0.16	9.27	20	0.02	14	5.6	420	23	260	N
30	−1.12	4	4	3.81	5.92	253	0.64	24	9.7	N/A	3.31	231.9	N

**Table 2:** Genetic analysis results of 30 children with *NR5A1* mutations.

ID	Nucleotide Mutation	Amino acid Mutation	Exon	Mutants From	ACMG classification	Domain
1	c.63C > T	p.S21F	Exon 2	*De novo*	Uncertain significance	DBD
2	c.86C > T	p.T29M	Exon 2	*De novo*	Pathogenic	DBD
3	c.274C > T	p.R92W	Exon 4	*De novo*	Pathogenic	DBD
4	c.319C > T	pQ107^∗^	Exon 4	*De novo*	Pathogenic	DBD
5	c.305-310del	p.104-105del	Exon 4	*De novo*	Pathogenic	DBD
6	c.645_c.646insG	p.P216Afs^∗^10	Exon 4	Mother	Pathogenic	Hinge Region
7	**c.937C > T**	**p.R313C**	Exon 5	*De novo*	Pathogenic	LBD
8	**c.937C > T**	**p.R313C**	Exon 5	*De novo*	Pathogenic	LBD
9	c.1052C > A	p.A351E	Exon 6	Mother	Pathogenic	LBD
10	c.1273delG	p.E425Rfs^∗^5	Exon 7	*De novo*	Pathogenic	LBD
11	c.1289G > T	p.S430I	Exon 7	*De novo*	Likely pathogenic	LBD
12	c.1236C > A	p.Cys412^∗^	Exon 7	Mother	Pathogenic	LBD
13	c.1138 + 1G > A	–	–	*De novo*	Pathogenic	–
14	c.245-2A > T	–	–	*De novo*	Pathogenic	–
15	c.982G > A	p.G328R	Exon 5	*De novo*	Pathogenic	LBD
16	3G > A	P.M1I	Exon 2	Mother	Pathogenic	DBD
17	c.1250delA	p.Q417Rfs^∗^13	Exon 7	*De novo*	Pathogenic	LBD
18	c.99C > A	p.C33X	Exon 2	Mother	Pathogenic	DBD
19	c.938G > A	**p.R313H**	Exon 5	*De novo*	Pathogenic	LBD
20	c.76G > A	p.G26R	Exon 2	*De novo*	Uncertain significance	DBD
21	247G > a	p.V83M	Exon 4	*De novo*	Uncertain significance	DBD
22	c.250C > T	p.R84C	Exon 4	Father	Likely pathogenic	DBD
23	**c.259C > T**	**p.R87C**	Exon 4	*De novo*	Likely pathogenic	DBD
24	**c.259C > T**	**p.R87C**	Exon 4	*De novo*	Likely pathogenic	DBD
25	**c.259C > T**	**p.R87C**	Exon 4	*De novo*	Likely pathogenic	DBD
26	c.614dupC	p.P206Tfs^∗^20	Exon 4	*De novo*	Pathogenic	Hinge Region
27	c.603T > A	p.Y201X	Exon 4	*De novo*	Pathogenic	Hinge Region
28	c.937C > T	**p.R313C**	Exon 5	*De novo*	Pathogenic	LBD
29	c.877G > A	p.D293N	Exon 5	*De novo*	Uncertain significance	LBD
30	c.756C > A	p.T252 =	Exon 4	*De novo*	Uncertain significance	LBD

*The bold values mean the most frequency mutations.*

### Hormone Measurements

The average ACTH level was 21.2 ± 7.1 pg/ml (normal, 0–46), and the average Cor level was 9.6 ± 3.1 μg/dl (normal, 5–25). The average basal LH/FSH ratio of the patients was 0.23 ± 0.29, and the basal LH and post-hCG T levels showed no differences between boys and girls (*p* > 0.05); however, the basal FSH level, LH/FSH ratio, and INHB level were lower in girls than in boys (*p* = 0.002; *p* = 0.001; *p* = 0.006) (Table 3). There were 21 patients in prepuberty, 1 patient in mini-puberty (his T level was 81.5 ng/dl), and 8 undergoing spontaneous puberty, with basal T concentrations ranging from 99.1to 289 ng/dl. The T concentrations were >100 ng/dl after hCG stimulation, except in 2 patients who had T concentrations lower than 20 ng/dl.

**Table 3:** Hormonal differences between boys and girls with *NR5A1* mutations.

Social gender	PG	Hight	T	LH	FSH	LH/FSH	INHB
Male	3	−0.33 SDS	282.87 ± 176.34	1.67 ± 1.56	7.84 ± 7.92	0.33 ± 0.26	121.38 ± 92.21
	IQR (2–3)	(median)					
Female	2	−0.68 SDS	278.05 ± 193.67	5.34 ± 7.12	22.95 ± 22.61	0.17 ± 0.12	38.89 ± 31.67
	IQR (3–4)	(median)					
*p*	0.086	0.384	*p* > 0.05	*p* > 0.05	0.002	0.006	0.001

### Molecular Analysis of the *NR5A1* Gene

There were 24/30 patients with *de novo* mutations, accounting for 80% of the affected patients. Five mutations were from the mothers, including the mothers of patients 16 and 18 who showed premature ovarian failure (menopause at 40 and 36 years). The ages of the other 3 mothers were 42, 28, and 38 years old, and these mothers experienced normal puberty and normal menses. Patient 22 inherited the mutation from a 23 years old asymptomatic father.

**FIGURE 1:**
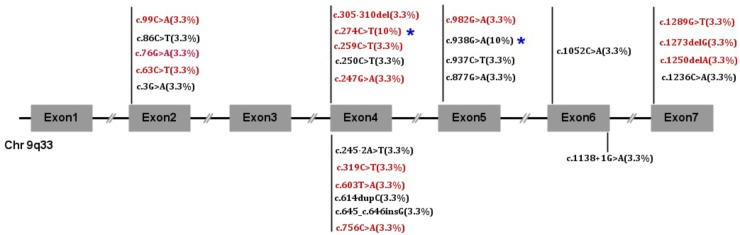
*NR5A1* mutation frequency in 30 children. Red font indicates novel mutations, and black font indicates reported mutations.

**FIGURE 2:**
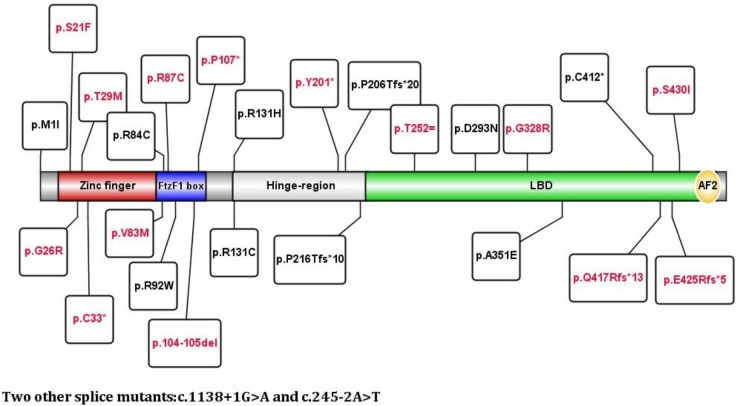
Relationship between mutations and the domain of the NR5A1 gene. Red font indicates novel mutations, and black font indicates reported mutations.

*NR5A1* genes were studied and showed 26 mutations. Twenty alleles were affected including 2 cuttings, 3 deletions and 2 insertions. Twelve are reported here: p.M1I, p.R84C, p.R92W, p.Q107^∗^, p.P206Tfs^∗^20, p.P216Afs^∗^10, p.D293N, p.R313H, p.R313C, p.A351E, p.C412^∗^, and c.1138 + 1G > A. Fourteen mutations have never been reported: p.S21F, p.G26R, p.T29M, p.C33^∗^, p.V83M, p.R87C, p.104-105del, p.Y201^∗^, p.T252=, p.G328R, p.Q417Rfs^∗^13, p.E425Rfs^∗^5, p.S430I, and c.245-2A > T. The mutations p.R87C and p.R313C accounted for 10% (3/30) of all mutations, and the rest of the mutations occurred only once each. We also found that exon 4 was the most commonly affected in 40% of patients. Exon 4 was affected in 72.7% of boys and 21% of girls, and exon 5 was the next most commonly affected (6/30). No mutations were found in exon 3. There were no differences in the clinical features between the DBD and LBD (*p* = 0.506). The detailed gene mutants are shown in Figures 1, 2.

### Follow-Up

The duration of follow-up was 2.96 ± 2.3 y. Sixteen of the 19 girls changed their genders to male, including five patients (patients 3, 7, 8, 11, and 17) who had spontaneous puberty. They had hypospadias repaired, including some after treatment with T undecanoate. Patients 16 and 19 used a GnRHa to inhibit gonadal development and waited until psychological maturation to make their own decisions regarding surgery at an appropriate time. Nine children were treated with T undecanoate to improve the appearance of a small penis. None of the patients underwent gonadectomy. Three girls’ parents tended to rear them as girls. One had no T response to hCG administration, another had a good T response, and another 11 years old patient had a basal T level >120 ng/dl. For these three children, we advised the parents against surgery until the patients could make their own decision and encouraged GnRHa treatment when necessary. Five of 11 boys had repaired hypospadias, and others had treatment for a small penis. All of them are now enjoying their gender.

## Discussion

DSD patients with *NR5A1* mutations have demonstrated phenotypic variability without a clear genotype–phenotype relationship since the first case was reported. The external genitalia can present as normal, ambiguous, severe hypospadias or a female type (Camats et al., 2012; Domenice et al., 2016). Additionally, several reports have shown patients with 46, XX DSDs have ovarian malformations or POI. Until now, no relationship has been established (Lourenco et al., 2009). This study also showed a variety of clinical phenotypes in 30 Chinese patients. Previous study for heterozygous NR5A1+/− mice only revealed adrenal insufficiency during stress conditions and showed significant adrenal hyperplasia, demonstrating that normal gene dosage of SF-1 is required for mounting an adequate stress response (Bland et al., 2000). Though none of our 30 patients had adrenal insufficiency, they may underlie subtle forms of subclinical adrenal insufficiency, which may become life threatening during traumatic stress. So it’s necessary to assess adrenal function regularly in order to make adequate preparations under stress.

The gonads were all testes without any residual Müllerian structures. Although the Prader stage at the first visit showed no difference between boys and girls, that may be mainly due to the fact that the decision about social gender by the parents is not always the best one and as well as these patients undergoing masculine development gradually after birth. This phenomenon also suggests that the Leydig cells may have considerable to be functional and can lead to virilization. This finding is consistent with previous research (Tantawy et al., 2012; Fabbri et al., 2014).

T concentrations were within the normal range in most patients, with mini-puberty or spontaneous puberty being displayed, and prepubertal patients had a good T response after hCG stimulation, except for two patients who likely had no remnant Leydig cells. We also observed remarkably increased FSH concentrations and a low LH/FSH ratio. The ratio was much lower in girls than in boys, which suggested that the female phenotype was a more severe type and that *NR5A1* mutations may more severely impair Sertoli cells than Leydig cells. S. Tantawy (Tantawy et al., 2012) observed similar results when examining gonadal function; Sertoli cell function gradually decreased with age, resulting in oligozoospermia or azoospermia. Decreased AMH can be associated with incomplete regression of Müllerian structure remnants with *NR5A1* mutations (Allali et al., 2011). In this group, patients had high FSH concentrations without Müllerian structures, demonstrating that there was enough AMH in the fetus to inhibit the growth of Müllerian structures. Whether or not patients develop Müllerian structures depends on the speed of attenuation of Sertoli cells. Therefore, as Tantawy suggested, Sertoli cell function decreases gradually with age (Allali et al., 2011; Tantawy et al., 2012). Two patients (patients 16 and 18) had a positive family history. The family members carried mutations associated with 46, XY DSDs or 46, XX premature ovarian failure. On the other hand, in some families, the carriers had no clinical manifestations, such as the father and mother of some patients in this study, which may be related to incomplete penetrance or young age (Philibert et al., 2007; Lourenco et al., 2009). We report there was a “temporary” family history that was negative or an unreliable negative family history because we noticed that in all the literature we cited above, the researchers only recorded the menstrual cycle of the mother but not the age of menarche or menopause. Therefore, it was difficult to evaluate whether there was true incomplete penetrance or ambiguous family history. The same is the case when the mutant is inherited from the father.

After checking the available databases, we did not find on relation of height and NR5A1 mutations carrier. Peycelon et al. (2017) reported that NR5A1 mutations can cause growth retardation before 1 year of age, and this gap in height increases with age. However, another study showed that the heights of patients with NR5A1 mutations were similar to those of their peers, especially among adolescent children, which may be associated with the secretion of T (Tantawy et al., 2012). In this study, the average height of the 30 children was lower than their target heights. The bone age was similar to that of normal children, indicating that T may play a role in growth and development, which is identical to the findings of Tantawy’s study (Tantawy et al., 2012).

Gender rearing for DSD patients has been a hot topic of debate. Most 46, XY girls with *NR5A1* mutations will undergo progressive masculinity if the gonads are not removed. As shown above, boys with *NR5A1* mutations can undergo spontaneous puberty (Tantawy et al., 2012; Fabbri et al., 2014). Additionally, patients with *NR5A1* mutations and preserved fertility have also been reported, suggesting that a 46, XY individual with an *NR5A1* mutation reared as a boy has certain advantages (Bashamboo et al., 2010; Philibert et al., 2011; Yagi et al., 2015); this finding was confirmed by the case of the father of one of our patients who had the same mutation as the patient but had preserved fertility. In this group, five mothers had the same mutation, and 2 of them had premature ovarian failure, indicating that 46, XX individuals with *NR5A1* mutations can have a natural pregnancy. The 3 other mothers, with an average age of 36 years, and one father were normal, but this finding must be followed up to determine if these individuals had premature failure. These facts suggest that an early sex change may be hasty. Caution should be taken when early gonadectomy is considered to lead to irreparable changes. In this study, with the exception of three patients whose parents insisted on rearing them as girls, all others underwent repair of hypospadias, were reared as boys and continued to do well until now. We therefore suggest that GnRHa protects gonadal function and inhibits the masculinization process until the child reaches psychological maturity.

*NR5A1* mutations are associated with a wide spectrum of gonadal development disorders, ranging from DSDs to oligo/azoospermia in 46, XY individuals (Camats et al., 2012; Domenice et al., 2016). In this study, 26 different mutations were found including 14 novel mutations. The mutations were mainly located in exon 4 and not in exon 3. This finding was different from those of previous studies in that the mutation sites were dispersed (Baetens et al., 2017; Rocca et al., 2018). The phenotypes of the reported mutations were similar to those previously published (Reuter et al., 2007; Allali et al., 2011; Camats et al., 2012; Yagi et al., 2015; Domenice et al., 2016; Fabbri et al., 2016; Baetens et al., 2017; Igarashi et al., 2017). We also identified 14 novel mutations that contributed to the improving our understanding on this subject.

The DBD region is crucial for transcription factors to bind to the DNA promoter and induce clinical expression. Studies have shown that mutations in the DBD may be more severe than mutations in other regions (Venselaar et al., 2010; Fabbri et al., 2016). However, in this group, we did not find that mutations in the DBD were more severe than those affecting the LBD. Changes in p.G26E, causing severe phenotypes, and p.T29R, leading to moderate phenotypes, have been reported (Domenice et al., 2016). In this study, patient 20 who had a p.G26R mutation and patient 2 who had a p.T29M mutation presented as Prader stage 2–3. This finding was different from those of previous research and may be related to the protein conformation. The variant p.C33^∗^ directly interacts with the first zinc finger protein. It is known that cysteine affects the 3-D structure of the whole protein; therefore, cysteine changes lead to an inability of DNA to interacts with the zinc finger, causing loss of the whole protein function. The patient only showed obvious masculinity at puberty with good testicular function. The Ftz-F1 box is considered a stabilizing region (DBD) for SF-1 binding to DNA and is highly conserved in different species (Little et al., 2006). The patients with p.V83M, p.R87C and p.104-105del mutations in the Ftz-F1 box motif presented as Prader stages 3-5 in this study. Thus, the clinical phenotype of the for patients with mutation in DBD domain remained to be investigated. The hinge region is an important domain for the interaction of the LBD with other proteins. Patient 27 had a moderate phenotype with the mutant p.Y201^∗^, leading to SF-1 losing the LBD region, which was consistent with the findings of previous studies (Baetens et al., 2017). LBD mutations may have varying effects depending on their location and alterations in ligand specificity/recognition (Domenice et al., 2016). The p.E425Rfs^∗^5 and p.Q417Rfs^∗^13 mutations result in a truncated protein that loses the AF-2 region, leading to a loss of protein function. The two patients who presented as Prader stage 0 and Prader stage 2 had relatively severe phenotypes. The p.S430I mutation located in the LBD C-terminus causes the loss of hydrogen bonds in the protein core and disrupts correct protein folding (Venselaar et al., 2010); one patient with this mutation presented as Prader stage 4. Therefore, the severity of the clinical phenotype caused by the *NR5A1* mutation site is not necessarily dependent on the affected domain. The c.245-2A > T mutation was not included in the ExAC and was not an SNP, indicating that it was a rare mutation. The one patient who presented with this mutation presented as Prader stage 0, indicating that this mutation may likely promote a prematurely truncated protein and lead to a severe phenotype. There were no clear relationships among the novel mutations. Functional studies are necessary to verify the pathogenicity of these novel mutations and should be carried out in the future.

### Limitations

First, the sample size may have been too small to establish a relationship between genotypes and phenotypes, and our future work will continue to increase the sample size to further focus on this topic. Second, the follow-up time can be extended to be longer period of time. Third, functional experiments will be needed to characterize the novel mutants, and we will perform these experiments in the following steps. Furthermore, compared to mass spectrometry, the use of a radioimmunoassay may be insufficient for detecting T levels. Lastly, only *AR* and *SRD5A2* were examined in some patients, and other genes related to 46, XY DSDs were excluded. Therefore, we will use a gene panel or next-generation sequencing (NGS)-based approach to make the diagnosis more comprehensive.

## Conclusion

Patients with 46, XY *NR5A1* mutations can clinically present with a wide spectrum of external genitalia and more severe Sertoli cell impairment than Leydig cell impairment. Mutations occurred often in exon 4. The novel p.R87C and reported p.R313C mutations appeared to be common (10%) in this group. The 14 new mutations enriched the mutation database and illuminated the particularity of Chinese people. The specific genotype–phenotype relationship remained to be established during future work.

## Author Contributions

CG conceivedand designed the study, provided critical comments and edited the manuscripts. YS analyzed the data and wrote the first draft. YS and LF collected the data. All authors read and approved the final manuscript.

## Conflict of Interest Statement

The authors declare that the research was conducted in the absence of any commercial or financial relationships that could be construed as a potential conflict of interest.The reviewer BP and handling Editor declared their shared affiliation.

## Footnotes

1 http://www.hgmd.cf.ac.uk/ac/gene.php?gene=NR5A1

2 http://genetics.bwh.harvard.edu/pph2/

3 http://sift.jcvi.org/

## Abbreviations

ACTH: adrenocorticotropic hormone
AMH: anti-Müllerian hormone
Cor: cortisol
DBD: DNA-binding domain
DSD: disorder of sex development
FSH: follicle-stimulating hormone
GnRHa: gonadotropin-releasing hormone agonist
hCG: human chorionic gonadotropin
INHB: inhibin B
LBD: ligand-binding domain
LH: luteinizing hormone
POI: premature ovarian insufficiency
SDS: standard deviation score
T: testosterone
